# Publication time valid prediction of citation risk outcomes in a bounded clinical specialty literature corpus

**DOI:** 10.21203/rs.3.rs-9772079/v1

**Published:** 2026-06-26

**Authors:** Sunny Chung, Charles Kahi, Siddharth Singh

**Affiliations:** Yale University; Indiana University School of Medicine, Roudebush VAMC; Mayo Clinic

**Keywords:** Citation prediction, bibliometrics, embeddings, citation network, semantics

## Abstract

Citation-prediction studies often estimate citation counts using information unavailable at publication. We evaluated whether citation-risk outcomes can be predicted using only publication-time information: metadata, references, author history, and text available on or before publication. We assembled 9,424 original-research articles published from 2017 to 2022 across seven clinical gastroenterology journals using OpenAlex and PubMed. The primary reference-observed cohort included 8,409 articles with a parsed reference list. The primary outcome was ≤ 3 citations within 2 years; secondary outcomes were 0 citations within 3 years, ≤ 3 citations within 3 years, and > 20 citations within 2 years. Models compared a nonsemantic citation/reference/context baseline, author-history variables, whole-document title/abstract embeddings, role-segmented source-text embeddings, and reference-context distributional features. Evaluation used two held-out publication-year folds with PR-AUC, or area under the precision-recall curve, F1, and precision among the top-ranked 10% of predictions. For the primary outcome, the nonsemantic baseline achieved PR-AUC 0.818, F1 0.722, and precision@10% 0.935. Adding whole-document embeddings improved performance to 0.828, 0.735, and 0.962, respectively. Structure-aware features did not improve the primary outcome but provided endpoint-specific gains for secondary outcomes. Author-history features showed standalone signal but did not improve the baseline. Pooled performance exceeded journal-local performance, indicating that citation-risk signal operated at the corpus level. These findings support publication-time-valid citation-risk modeling as a reproducible framework for studying evidence visibility within bounded literatures and motivate replication across other journal sets, specialties, and publication eras.

## Introduction

Citation prediction is often framed as estimating future impact, but its scientometric value depends on whether future citation visibility can be inferred from information available when a paper enters the literature. This distinction matters because citation outcomes are not direct measures of scientific merit. Within bounded specialty literatures, they are better interpreted as observable traces of how evidence becomes visible, remains peripheral, or accumulates attention. Papers may become effectively uncited, remain in a low-citation band, or rapidly accumulate early attention. Because citation distributions are heavy-tailed and early visibility can compound over time, a publication-time design requires endpoints that are interpretable without using post-publication citation information as predictors (Penner et al., 2013; Wang et al., 2013).

Prior citation-prediction studies have used structured bibliographic features (Yan et al., 2011), impact-factor and early-citation quantiles (Stegehuis et al., 2015), neural network and deep learning frameworks (Abrishami & Aliakbary, 2019), citation-based impact learning frameworks (Weihs & Etzioni, 2017), and dynamic citation-network models (He et al., 2023).

These approaches rest on a shared interpretive premise: citations measure visibility and cumulative attention rather than intrinsic scientific quality. Citation indexing was originally proposed as a way to organize scientific literature through reference links (Garfield, 1955); cumulative advantage theory explains why early visibility and prior attention can compound over time (Price, 1976); and studies of citation behavior show that citations reflect heterogeneous processes of attention, use, persuasion, visibility, and social structure (Bornmann & Daniel, 2008; Tahamtan & Bornmann, 2018). Because citation indicators require normalization and contextual interpretation before evaluative use (Waltman, 2016), citation-prediction models should be evaluated as bounded models of visibility or attention.

Several design choices limit prospective interpretation. Random rather than temporal partitions can allow models to exploit feature distributions or citation structures unavailable at the intended prediction time. Feature pipelines can also introduce information from the evaluation period into training, creating leakage, a problem described more broadly in machine-learning-based science (Kapoor & Narayanan, 2023). Outcome definitions are often chosen for modeling convenience rather than interpretability, so apparently strong models may not correspond to meaningful citation-risk states. Broad field-agnostic corpora can support benchmark claims but may obscure how prediction behaves within bounded specialty literatures, where topics, venues, and citation practices are locally organized. These concerns parallel prediction-model reporting principles, including prespecified endpoints, time-valid predictor construction, and validation aligned with intended use (Collins et al., 2015).

Recent infrastructure makes such a model design feasible. OpenAlex provides an open index of scholarly works, authors, venues, institutions, concepts, and citation links (Priem et al., 2022), with recent-publication reference coverage comparable to Web of Science and Scopus for several metadata elements (Culbert et al., 2025). Scientific text representation models can also encode title, abstract, and structured document signals using representations trained for scientific literature (Cohan et al., 2020; Singh et al., 2023). These resources jointly support citation-prediction designs that combine publication-time metadata, reference structure, author history, and semantic information while preserving temporal validity.

We therefore evaluated citation prediction as a publication-time-valid prediction problem, asking three questions: whether citation-risk outcomes can be predicted from information available at publication; whether text-embedding and reference-context features add signal beyond citation and reference structure alone; and whether pooled multi-journal performance translates to journal-local validity. Predictors were restricted to metadata, references, author history, and article text available on or before each source paper's publication date. We compared four predictor groups: a nonsemantic citation/reference/context baseline, author-history features, whole-document title/abstract embeddings, and structure-aware additions.

## Methods

### Study design

We conducted a retrospective, time-indexed prediction study designed to emulate citation prediction at the time an article entered the literature. The unit of analysis was the source paper. Predictors were restricted to information available on or before the source paper’s publication date. The study therefore asked whether future citation-risk outcomes could be predicted from publication-time signals, not whether citation counts could be retrospectively ranked after later information became available.

We applied leakage-control rules before model comparison. These rules were intended to prevent information from the validation or test period from entering predictor construction. They included preserving all source-paper rows during author-feature merges, avoiding pre-split imputation of author-history features, excluding same-year prominence variables, excluding metadata fields not reliably available at publication time, and applying explicit citation-outcome rules. Additional leakage-control details are provided in **Online Resource 1**.

### Corpus assembly and eligibility

Corpus construction had three steps: identifying candidate articles from seven masked clinical gastroenterology journals, applying eligibility and source-record exclusions to retain original research articles, and defining the analysis cohorts according to reference-list availability. The analytic corpus consisted of article-type papers published from 2017 through 2022 across seven clinical gastroenterology journals, identified throughout the manuscript by stable masked codes Journal A through Journal G. Journal identities were masked because the inferential target was publication-time-valid prediction in a bounded specialty corpus, not comparison or ranking of named journals. All analyses used stable masked journal identifiers for corpus construction, feature derivation, temporal splitting, endpoint labeling, and held-out prediction summaries.

Five journals entered the corpus directly through OpenAlex-derived article datasets containing work-level bibliographic metadata, authorship, cited-work identifiers, publication dates, journal metadata, abstracts, and yearly citation trajectories. Reference-level metadata for cited works were obtained from local metadata aligned to the same OpenAlex work datasets. For source-text embedding features, source-paper abstracts preferentially used PubMed-enriched abstracts when available; reconstructed OpenAlex abstract fields were used when PubMed abstracts were unavailable. Journals E and G required a shared clinical-corpus preprocessing branch because their source export included both clinical gastroenterology articles and basic science or translational research articles; this preprocessing is described in detail in **Online Resource 2**.

Within the Journal E/G preprocessing branch, deterministic publication-type, Medical Subject Headings (MeSH), and title rules resolved clear cases first. Records remaining unresolved after deterministic triage were classified using a prompt-constrained large language model triage step; 3,020 records were submitted to the large language model, of which 1,669 were assigned include, 1,334 exclude, and 17 uncertain before audit. A stratified audit of 206 records identified 35 disagreements; deterministic rules were revised where applicable, and corrected classifications were used before modeling.

Eligibility criteria were applied reproducibly. We retained records classified as articles and published between 2017 and 2022, inclusive. Before final article-level filtering, we applied prespecified source-record exclusions for guideline-like titles and journal-specific abstract or supplement DOI patterns. These exclusions removed records that carried article-type metadata but did not represent original research articles. The exact title and DOI rules are provided in **Online Resource 3**. The DOI-pattern exclusions removed 5,210 source records, predominantly abstract or supplement records, including 4,475 from Journal A and 735 from Journal D.

After eligibility filtering, the final analytic corpus included 9,424 papers. Because reference-derived predictors can only be interpreted when a paper has an available parsed reference list, we defined two analysis cohorts. The primary cohort was the reference-observed cohort, consisting of 8,409 papers with a non-empty reference list. The full analytic corpus was retained as a sensitivity cohort to evaluate whether the reference-observed filter over-narrowed the corpus and to quantify the practical effect of incomplete bibliography coverage.

**Figure 1.**
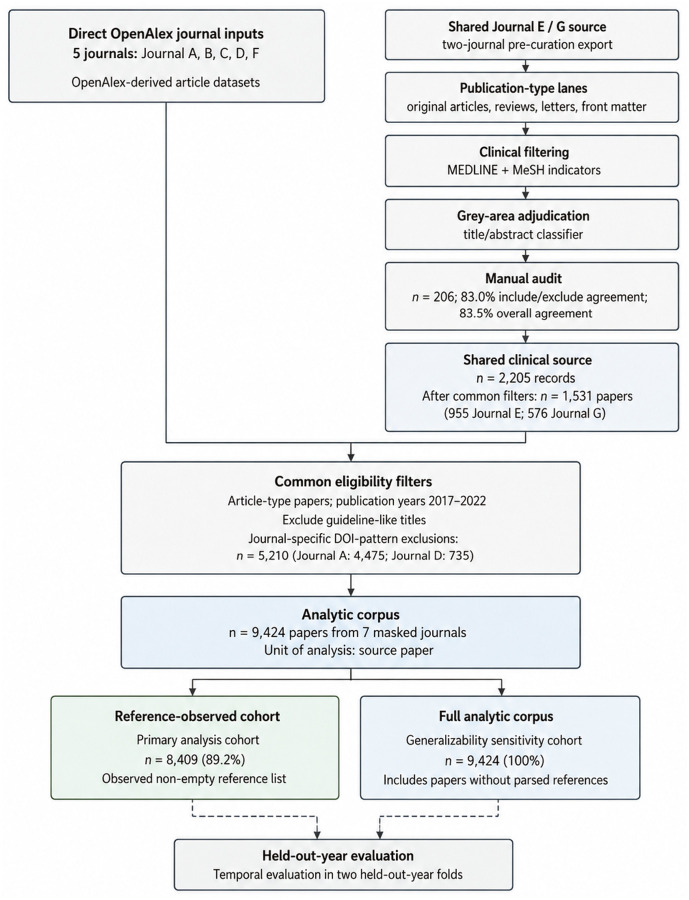
Study design, corpus assembly, and analytic cohorts Flow diagram showing the journal inputs (masked codes Journal A through Journal G), the additional clinical-corpus curation branch for Journal E and Journal G, the abstract/supplement DOI exclusions for Journal A and Journal D, the common article and guideline-like title filters, separation into the full analytic cohort and the reference-observed cohort, endpoint-specific availability for 3-year and 2-year outcomes, and the two held-out-year temporal evaluation folds

Figure 1 summarizes the study-design flow, including journal inputs, the Journal E/G preprocessing branch, common eligibility filters, full and reference-observed cohorts, endpoint-specific availability, and the two held-out-year evaluation folds.

### Citation outcomes

The primary endpoint was low short-term citation visibility, defined as ≤ 3 citations within 2 years. This endpoint was selected because it was common enough to model reliably and aligned with the 2-year window used in common journal citation metrics. Secondary endpoints captured adjacent citation-risk states: complete noncitation, defined as 0 citations within 3 years; broader low 3-year visibility, defined as ≤ 3 citations within 3 years; near-complete invisibility, defined as ≤ 1 citation within 3 years; and high short-term citation accumulation, defined as > 20 citations within 2 years.

Three-year outcomes used the precomputed 3-year citation-count field from the journal-source citation records in OpenAlex. Two-year citation counts were computed from yearly OpenAlex citation trajectories as calendar-year counts accrued in the publication year and the following calendar year. Papers without valid yearly citation trajectories were excluded from 2-year endpoint analyses. Two-year endpoints were available for 8,711 of 9,424 papers in the full analytic cohort and for 7,988 of 8,409 papers in the reference-observed cohort.

### Predictor families

Predictors were organized by the type of publication-time information they represented. The nonsemantic citation/reference/context baseline included publication context, such as masked journal and publication year; reference-list composition, such as reference age and reference type; cited-knowledge maturity, defined as citation and visibility characteristics of the papers cited by the source paper as of the source paper’s publication date; and ancestry/novelty features, defined as measures of how the source paper recombined prior literature and two-hop backward citation lineages.

Author-history variables summarized prior author citation history and collaboration-network context using only information available before the source paper’s publication date. These features were evaluated empirically rather than assumed to be useful. For the complementary author-history assessment, a tertile-derived low-versus-not-low citation outcome was used rather than the absolute citation-risk endpoints applied to the main model families. This operationalization was chosen to isolate whether author-history features add predictive information beyond the nonsemantic baseline under a balanced classification task; it was not treated as a primary citation-risk endpoint because relative citation buckets are less directly interpretable than absolute thresholds.

Text-based semantic features and reference-context features were treated as related but methodologically distinct predictor blocks. Whole-document semantic features used SPECTER2 embeddings of the source paper’s title and abstract treated as a single text block. Structure-aware content features used SPECTER2 embeddings of source-paper title/abstract text split into prespecified role-style segments: opening, body, closing, and summary. Structure-aware contextual features did not embed text directly; instead, they represented each source paper’s cited-literature context as a publication-time distribution over cited source/journal identifiers and compared that distribution with year-specific low- and not-low-citation reference-context centroids estimated within the training fold from prior eligible papers. The combined content-plus-context model included both the role-segment content features and the reference-context distributional features.

The resulting model families were the nonsemantic baseline, the whole-document semantic model, the structure-aware content model, the structure-aware contextual model, and the combined structure-aware content-plus-context model. The whole-document semantic model served as the semantic comparator. Structure-aware models were evaluated as endpoint-specific alternatives, not as a strict ordered ladder. The mapping from manuscript family labels to archived stage labels is provided in **Online Resource 4**.

### Model development and temporal evaluation

Models were evaluated using temporal rather than random splits. In the first fold, models were trained on 2017–2019 publications, tuned on 2020 publications, and tested on 2021 publications. In the second fold, models were trained on 2017–2020 publications, tuned on 2021 publications, and tested on 2022 publications. This design approximated sequential prospective use: each test year was evaluated only with models trained and tuned on earlier years.

Within each temporal fold and endpoint, models were trained as supervised binary classifiers using the prespecified feature-family stages. Preprocessing was fit within the training years and then applied unchanged to the validation and held-out test years. Numeric features were median-imputed. Categorical features were most-frequent-imputed and one-hot encoded, with unseen validation or test categories ignored. For semantic stages, dimensionality reduction was estimated within the training years of each fold only, preventing validation-year or test-year information from influencing the semantic representation used in that fold.

We evaluated two learner families: regularized logistic regression and gradient-boosted trees. Regularized logistic regression used an elastic-net penalty. The regularization strength and elastic-net mixing parameter were selected from a fixed validation-year grid within each fold. Gradient-boosted tree models used XGBoost. Hyperparameters were selected from a fixed validation-year grid spanning tree depth, learning rate, subsampling, column sampling, minimum child weight, L1/L2 regularization, and number of boosting rounds. Both learner families were eligible for endpoint-specific selection.

All stochastic model components and fold-local dimensionality-reduction steps used a fixed random seed. No preprocessing step was fit on the pooled dataset before temporal splitting. This ensured that feature scaling, imputation, encoding, semantic dimensionality reduction, hyperparameter selection, and threshold selection were all restricted to the appropriate temporal fold.

### Metrics, uncertainty, and endpoint-specific reporting selection

Performance was summarized using three primary metrics. PR-AUC, or area under the precision-recall curve, measured ranking performance and was emphasized because several endpoints were imbalanced. F1 measured thresholded classification performance at the validation-selected operating point. Precision@10% measured the proportion of true positive cases among the top-ranked 10% of held-out predictions, providing a fixed-depth ranking summary. These metrics capture different properties: PR-AUC reflects overall ranking, F1 reflects a chosen decision threshold, and precision@10% reflects top-decile enrichment.

Probability thresholds for binary classification were chosen within each temporal fold on that fold’s validation year by maximizing validation F1. The selected thresholds were then applied without re-selection to the held-out test year for that fold.

Reported pooled held-out metrics were computed over the concatenation of the 2021 and 2022 test rows from the two temporal folds. In the reference-observed cohort, this corresponded to 2,851 held-out rows for the 3-year endpoints and 2,626 held-out rows for the 2-year endpoints. The 2-year basis was smaller because those endpoints depended on recovered yearly citation trajectories. We used a top-decile precision threshold rather than a fixed k because held-out denominators differed across 2-year and 3-year endpoints.

Bootstrap intervals were computed from archived held-out predictions rather than from full retraining resamples. This pragmatic choice holds trained models fixed and resamples only held-out evaluation rows; therefore, it reflects evaluation-row sampling variance but understates variance attributable to model fitting. For each endpoint, we report fold-stratified bootstrap intervals for retained-model performance and paired fold-stratified bootstrap intervals for retained-versus-comparator metric differences. The paired analysis resampled the same held-out rows, stratified by fold and label, to isolate model-pair differences from fold and label noise. Bootstrap procedures are described in **Online Resource 6**.

We designated an endpoint-specific retained family using a prespecified multi-metric reporting heuristic. A structure-aware feature family was retained over the whole-document semantic comparator only when PR-AUC improved by at least 0.005, thresholded F1 did not decrease by more than 0.002, and precision@10% did not decrease by more than 0.01. The heuristic was used only to select a compact reported family and was not treated as a hypothesis test; paired bootstrap intervals were used for inferential interpretation, and conclusions do not rely on the heuristic alone. Comparator and retained-family metrics are reported for each endpoint, with paired bootstrap intervals used to evaluate selected-versus-comparator differences.

The primary endpoint, temporal folds, reference-observed primary cohort, main feature-family ladder, retained-family reporting heuristic, and Journal C local-diagnostic design were specified before final endpoint-level model comparisons were conducted.

### Journal-local validity diagnostic

Primary performance summaries were computed in pooled cross-journal analyses. We additionally included a prespecified journal-local diagnostic for one within-corpus journal, Journal C. In this analysis, the pooled endpoint models were applied to held-out Journal C rows and evaluated under both the pooled default interpretation and local reinterpretations based on threshold adjustment or recalibration. Journal C was selected a priori because it was a high-volume source with incomplete but substantial reference coverage, enough held-out papers to support endpoint-specific local summaries, and a citation-prevalence profile that made local operating-point assessment feasible. The analysis was intended to demonstrate the need for local validation, not to characterize all journal-specific behaviors in the corpus. Details of the journal-local thresholding and calibration audit are provided in **Online Resource 7**.

## Results

Results are presented in the order of the analytic workflow: corpus characteristics and endpoint availability, the complementary author-history assessment, feature-family comparisons across citation-risk endpoints, uncertainty analyses, and the prespecified journal-local diagnostic.

### Corpus characteristics and endpoint availability

After fixed eligibility filtering, including the abstract and supplement DOI exclusions described in the Methods, the analytic corpus included 9,424 article-type papers published from 2017 through 2022 across seven masked clinical gastroenterology journals, identified as Journal A through Journal G. Of these, 8,409 papers (89.2%) had an observed non-empty reference list and entered the primary reference-observed analysis. Reference-list availability varied across journals, ranging from 77.1% in Journal A and 79.6% in Journal C to 92.9% in Journal D, 94.1% in Journal E, 99.5% in Journals B and F, and 100.0% in Journal G. Corpus composition by source and year is summarized in Table 1, and the study-flow diagram is shown in Fig. 1.

Endpoint prevalence varied by task and analytic cohort. For 2-year outcomes, valid yearly citation trajectories were available for 7,988 of 8,409 reference-observed papers (95.0%). In this eligible subset, the primary endpoint of ≤ 3 citations within 2 years had a prevalence of 38.0% (3,034/7,988), while the exploratory high-citation endpoint of > 20 citations within 2 years had a prevalence of 11.8%. Among all 8,409 reference-observed papers, the stringent complete-noncitation endpoint of 0 citations within 3 years was present in 648 papers (7.7%), the robustness endpoint of ≤ 1 citation within 3 years was present in 1,204 papers (14.3%), and the broader low-citation endpoint of ≤ 3 citations within 3 years was present in 1,987 papers (23.6%). In the full analytic corpus, 2-year outcomes were available for 8,711 of 9,424 papers (92.4%). These differences required endpoint-specific analytic denominators, particularly for comparisons between 2-year and 3-year outcomes. Endpoint availability and prevalence in both analytic cohorts are summarized in Table 2.

Masked-journal characteristics showed substantial within-corpus heterogeneity. Median 3-year citations ranged from 2 to 42 across journals, and primary-endpoint prevalence ranged from 7.5% to 76.3%. Masked-journal characteristics, including endpoint prevalence, median citation and reference counts, PubMed abstract coverage, and source branch, are reported in **Online Resource 8**; a curated-branch prevalence sensitivity is shown in **Online Resource 9**. In that prevalence-only sensitivity, excluding the curated Journal E/G branch increased low-citation endpoint prevalence and reduced high-citation endpoint prevalence, reinforcing that endpoint frequencies were source-branch dependent. This heterogeneity indicates that the corpus was bounded by specialty and source selection, not by a homogeneous citation ecology, and motivated the journal-local validity diagnostic.

### Author-history features had standalone but redundant signal

The main endpoint analyses used absolute citation-risk outcomes. The author-history analysis was separate and complementary. Its purpose was narrower: to test whether author-history features added predictive information beyond the nonsemantic citation/reference/context baseline. For this assessment, we used a tertile-derived low-versus-not-low outcome in the final cleaned reference-observed cohort rather than one of the absolute citation-risk endpoints.

Author-history features carried predictive signal but did not improve performance beyond the nonsemantic baseline. In the complementary tertile-derived low-versus-not-low task, evaluated in the final cleaned reference-observed cohort of 8,409 papers, the citation/reference/context baseline achieved F1 0.585 and balanced accuracy 0.682. Adding the full author-history feature block lowered F1 to 0.569, a decrease of 0.016 relative to the baseline; the lighter pruned author-history variant achieved F1 0.575, a decrease of 0.011. Author variables were nevertheless informative in isolation: an author-only model achieved F1 0.513, and adding publication-time metadata increased F1 to 0.573. These comparisons indicate that author-history features captured real citation-risk signal, but did not add incremental predictive value after conditioning on publication-time citation, reference, and structural context.

### Feature-family progression across citation-risk endpoints

We first assessed how much signal was captured by the nonsemantic citation/reference/context baseline. We then asked whether whole-document title/abstract embeddings improved performance beyond this baseline. Finally, we evaluated two structure-aware additions against the whole-document semantic comparator: role-segmented source-text embeddings and reference-list context features. Because these additions separated source-text representation from reference-context distributional signal, they were interpreted as endpoint-specific alternatives rather than as a single uniformly superior model class. The nonsemantic baseline, whole-document semantic comparator, and retained endpoint-specific family for each main endpoint are reported in Table 3.

For the primary endpoint, ≤ 3 citations within 2 years, the nonsemantic baseline already showed substantial corpus-level signal, with PR-AUC 0.818, F1 0.722, and precision@10% 0.935. The whole-document semantic model added a modest increment, with PR-AUC 0.828, F1 0.735, and precision@10% 0.962. This whole-document semantic model was retained because no structure-aware family met the prespecified reporting heuristic. The primary endpoint is therefore best interpreted as a whole-document semantic result rather than as evidence that structure-aware content/context features improved ranking or thresholded classification. Post hoc operating-point diagnostics for the retained primary model are reported in **Online Resource 10**.

For the stringent 0 citations within 3 years endpoint, the nonsemantic baseline achieved PR-AUC 0.436, F1 0.408, and precision@10% 0.434. Whole-document semantics improved PR-AUC and precision@10% but lowered F1. The combined content-plus-context model improved thresholded F1 from 0.325 to 0.453 relative to the whole-document semantic comparator, without improving PR-AUC or precision@10%.

For ≤ 3 citations within 3 years, the nonsemantic baseline performed similarly to the whole-document semantic comparator, with PR-AUC 0.858 versus 0.860. The retained structure-aware content model added small increments, reaching PR-AUC 0.866, F1 0.811, and precision@10% 0.948. This retained-family result should be interpreted cautiously: the PR-AUC increment over the whole-document semantic comparator was small, and paired bootstrap intervals for PR-AUC, F1, and precision@10% all spanned zero.

For the exploratory high-citation endpoint of > 20 citations within 2 years, the nonsemantic baseline achieved PR-AUC 0.452, F1 0.479, and precision@10% 0.460. Whole-document semantics improved top-decile ranking but not thresholded F1. The retained structure-aware contextual model improved PR-AUC to 0.509 with little change in precision@10%. For this endpoint, semantic features appeared to improve ranking signal relative to the whole-document comparator, but neither semantic model recovered the nonsemantic baseline’s thresholded F1, indicating a tradeoff between ranking performance and the validation-selected operating point.

**Figure 2.**
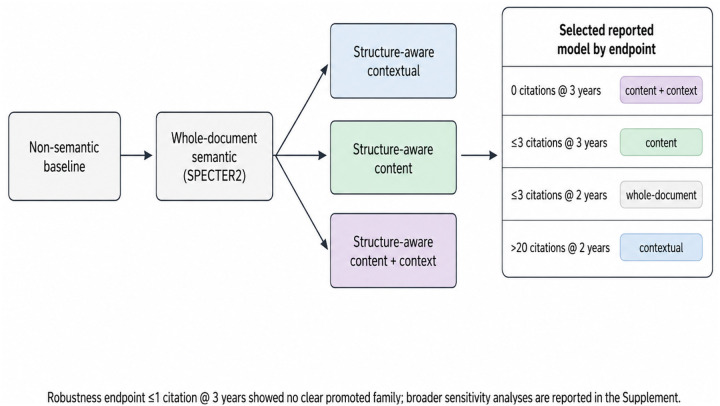
Model-family progression and endpoint-specific selection Conceptual comparison ladder used in the main analyses. The nonsemantic citation/reference/context baseline is reported as the feature-family reference point; whole-document semantics are then evaluated as the semantic comparator; endpoint-specific structure-aware feature alternatives are evaluated against the whole-document semantic comparator

**Figure 3.**
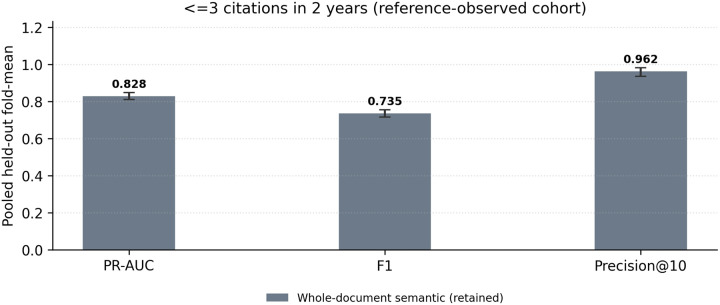
Primary endpoint results for <=3 citations in 2 years Performance of the retained primary endpoint family for ≤3 citations within 2 years in the reference-observed cohort, emphasizing PR-AUC, F1, and precision@10%. Bars show pooled held-out means with the corresponding 95% bootstrap intervals (as tabulated in **Online Resource 11**)

**Figure 4.**
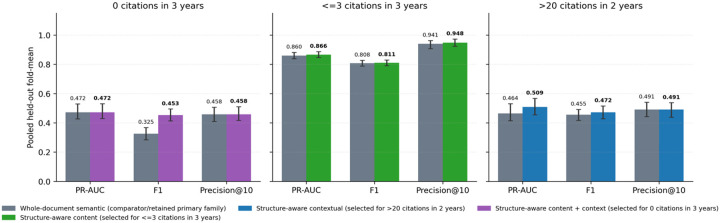
Secondary editorial endpoints Performance summaries for the modeled secondary endpoint comparisons: 0 citations in 3 years, 3 citations or fewer in 3 years, and more than 20 citations in 2 years. The 1-citation-or-fewer robustness endpoint is reported in the endpoint-prevalence table rather than in this model-comparison figure

For the ≤ 1 citation within 3 years robustness endpoint, semantic features improved performance over the nonsemantic baseline, but the structure-aware feature families did not yield a clear retained-family claim. In held-out reference-observed folds, the nonsemantic baseline achieved PR-AUC 0.640, F1 0.682, and precision@10% 0.703; the whole-document semantic model achieved PR-AUC 0.661, F1 0.694, and precision@10% 0.710. The highest structure-aware PR-AUC was observed for the content model at 0.664, below the prespecified + 0.005 reporting threshold versus whole-document semantics (paired ΔPR-AUC + 0.003; 95% bootstrap interval − 0.028 to + 0.032). Full point estimates and bootstrap intervals are reported in Online Resource 11. This endpoint was therefore retained as a robustness reference but excluded from the retained-family summary in Table 3. Model-family progression is shown in Fig. 2, the primary ≤ 3 citations within 2 years endpoint is shown in Fig. 3, and the secondary absolute citation-risk endpoints are shown in Fig. 4.

### Uncertainty estimates and paired model comparisons

Bootstrap intervals computed from archived held-out predictions are reported in **Online Resource 11**. For the primary ≤ 3 citations within 2 years endpoint, the retained whole-document semantic model achieved PR-AUC 0.828 (95% bootstrap interval, 0.809–0.847), F1 0.735 (0.716–0.754), and precision@10% 0.962 (0.935–0.981). For the stringent 0 citations within 3 years endpoint, the retained combined structure-aware content-plus-context model achieved fold-mean PR-AUC 0.472 (0.429–0.530), F1 0.453 (0.413–0.494), and precision@10% 0.458 (0.417–0.510). For ≤ 3 citations within 3 years, the retained structure-aware content model achieved PR-AUC 0.866 (0.845–0.886) and F1 0.811 (0.791–0.830). The exploratory high-citation endpoint remained less sharply separated, with the retained structure-aware contextual model achieving PR-AUC 0.509 (0.455–0.567) and F1 0.472 (0.428–0.515).

Paired fold-stratified bootstrap intervals were used to isolate model-pair differences on the same resampled held-out rows. These paired comparisons are also reported in **Online Resource 11**. For the primary ≤ 3 citations within 2 years endpoint, the whole-document semantic model was both the comparator and the retained family; therefore, the retained-versus-comparator delta was zero by construction. For the stringent 0 citations within 3 years endpoint, paired PR-AUC and precision@10% differences spanned zero: PR-AUC delta − 0.000 (95% paired bootstrap interval, − 0.029 to + 0.032) and precision@10% delta + 0.000 (− 0.035 to + 0.053). In contrast, paired F1 improved by + 0.128 (+ 0.087 to + 0.170), indicating a threshold-quality gain for this rare-event endpoint.

For ≤ 3 citations within 3 years, paired metric differences were small: PR-AUC delta + 0.006 (− 0.001 to + 0.013), F1 delta + 0.003 (− 0.006 to + 0.012), and precision@10% delta + 0.007 (− 0.011 to + 0.038). For > 20 citations within 2 years, none of the paired metric deltas excluded zero at the 95% level: PR-AUC + 0.045 (− 0.007 to + 0.089), F1 + 0.017 (− 0.023 to + 0.058), and precision@10% +0.000 (− 0.049 to + 0.042). The paired comparisons therefore support an endpoint-specific interpretation of structure-aware feature gains and an exploratory interpretation of the high-citation endpoint.

Because COVID-era publication and citation dynamics could affect endpoint prevalence, we added a descriptive publication-era sensitivity. For the primary ≤ 3 citations within 2 years endpoint in the reference-observed cohort, prevalence was 37.6% among 2017–2018 publications whose 2-year citation windows ended before 2020, 37.2% among 2017–2019 pre-pandemic publication years, and 38.6% among 2020–2022 publication years. This reduces concern that the primary endpoint definition was driven by COVID-era prevalence shifts, although it does not rule out era-related changes in feature-outcome relationships.

### Within-corpus local validity diagnostic

Pooled cross-journal performance did not translate directly into journal-local performance. In the prespecified Journal C diagnostic, the same endpoint models generally performed worse than in the pooled held-out analysis, showing that corpus-level signal should not be interpreted as local validity without journal-specific evaluation. Applying the pooled endpoint models to held-out Journal C papers yielded PR-AUC/F1/precision@10% of 0.205/0.326/0.145 for 0 citations within 3 years, 0.474/0.446/0.491 for ≤ 3 citations within 3 years, 0.696/0.645/0.815 for ≤ 3 citations within 2 years, and 0.240/0.247/0.259 for > 20 citations within 2 years.

For the low-citation endpoints, local thresholding did not materially improve F1 over the pooled default or produced only a near-tie. Local recalibration improved Brier score for some endpoints but did not necessarily improve thresholded classification. The largest journal-local change occurred for the exploratory high-citation endpoint: for > 20 citations within 2 years, local threshold and calibration selection improved F1 from 0.247 to 0.286 without improving ranking. These findings indicate that recalibration, threshold adjustment, and ranking quality are distinct properties of journal-local model behavior.

**Figure 5.**
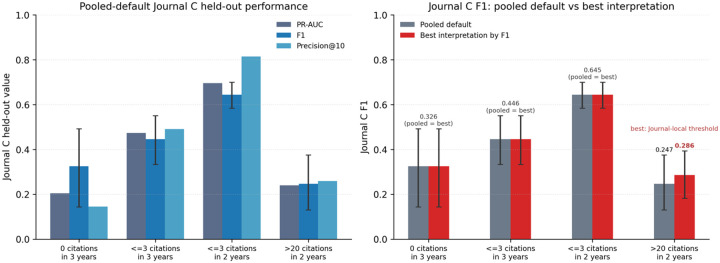
Pooled versus Journal C-local interpretation Comparison of pooled corpus-level performance with held-out journal-local performance in Journal C (the prespecified within-corpus journal retained for journal-local analysis), including the effect of local threshold adjustment for the high-citation endpoint. Threshold-adjusted results are highlighted where they differ materially from the pooled default

The pooled-versus-Journal C comparison and the effect of local thresholding are shown in Fig. 5, with detailed numeric comparisons provided in **Online Resource 5**. Because journal-local event counts differed by endpoint, **Online Resource 5** reports the Journal C eligible denominator, positive-label count, and prevalence alongside local performance metrics. Sparse local positive counts for 0 citations within 3 years (9 events) and > 20 citations within 2 years (43 events) support interpreting these endpoint-specific local results as unstable diagnostics rather than local validation claims. Overall, pooled multi-journal performance should be interpreted as corpus-level evidence of citation-risk signal, not as evidence of journal-local validity without local evaluation.

## Discussion

Citation counts are probabilistic, context-bounded outcomes rather than deterministic indicators of scientific value (Wang et al., 2013). Apparent predictability of future impact can also be shaped by cumulative advantage, autocorrelation, and career stage (Penner et al., 2013). Citation-risk models should therefore be understood as bounded diagnostics of visibility and attention, not as measures of epistemic value, methodological rigor, clinical importance, or translational significance.

Information available at publication contained measurable signal for low short-term citation visibility: for the primary endpoint of ≤ 3 citations within 2 years, the retained whole-document semantic model improved modestly over the nonsemantic citation/reference/context baseline. Structure-aware feature families showed endpoint-specific value, especially for thresholded classification of complete noncitation, but they did not provide a general improvement across all endpoints or metrics.

The broader contribution is methodological. In a bounded specialty corpus, citation-risk prediction could be evaluated under leakage-clean temporal validation, but no single endpoint, feature family, or pooled performance estimate established local readiness. Author-history features were predictive but nonincremental; structure-aware content/context features showed endpoint-specific additive signal; and corpus-level performance did not transfer to individual journals without local evaluation. The central implication is that citation-prediction claims depend on endpoint definition, feature-family decomposition, and validation setting. Under these constraints, publication-time-valid modeling can help characterize how content, reference ancestry, author history, semantic positioning, and venue context jointly shape whether evidence becomes visible.

### Temporal validity and leakage control

The leakage-control measures applied in this study, including within-fold preprocessing, held-out-year evaluation, and explicit citation-outcome rules, align citation prediction with broader principles of prediction-model transparency established in clinical research (Collins et al., 2015) and with leakage-control frameworks developed for machine-learning-based science more broadly (Kapoor & Narayanan, 2023; McDermott et al., 2021). The practical implication is that citation-prediction models should be judged not only by their reported metrics but by whether their feature construction, outcome definition, and validation design preserve the prospective interpretation claimed for the model. Studies that use random splits, pre-split imputation, or post-publication citation counts as predictors cannot support publication-time inference regardless of how strong their held-out performance appears.

### Author features carry real but redundant signal

In the complementary author-signal assessment, author-history variables did not improve the nonsemantic citation/reference/context baseline. This finding is consistent with prior work showing that author productivity and collaboration context are associated with future citation outcomes (Acuna et al., 2012; Sinatra et al., 2016), while also aligning with cautions that some apparent author-level predictability reflects autocorrelation in cumulative measures such as the h-index and varies by career stage (Penner et al., 2013).

This result should not be interpreted as showing that author information is irrelevant. Rather, in this bounded specialty corpus, much of the author-history signal relevant to citation-risk prediction was not incrementally expressed after conditioning on publication-time citation, reference, and contextual features. Reference composition, cited-knowledge maturity, journal/year context, and local structural features may overlap with, proxy for, or absorb prior-visibility signal captured by author-centric variables. Author priors should therefore be treated as empirically testable feature families rather than assumed necessities.

### Structure-aware features provided endpoint-specific additive signal

The retained feature family varied by endpoint. A plausible interpretation is that scientific text representations capture different forms of semantic positioning depending on the endpoint: document-level topic location, source-paper framing, and reference-context alignment may matter differently for noncitation, low-citation risk, and high short-term citation accumulation. Prior work supports this possibility, showing that scientific text representations can capture document-level relatedness and task-relevant structure beyond manually engineered variables (Cohan et al., 2020; Singh et al., 2023), and that citation-informed or neighborhood-structured representations can capture relatedness signals that isolated whole-document embeddings may miss (Ostendorff et al., 2022). Complete short-term noncitation, broader low-citation risk, and short-horizon high-citation emergence are therefore not interchangeable outcomes.

Secondary endpoint gains should be interpreted as endpoint-specific model-comparison findings rather than confirmatory evidence of a general structure-aware feature advantage. Retained families were selected separately by endpoint using a multi-metric heuristic, and paired bootstrap intervals did not exclude zero for the two endpoints where structure-aware models were favored on ranking metrics. The practical implication is that structure-aware feature decomposition is a useful analytic tool for characterizing endpoint-specific visibility signals, not a uniformly superior representation strategy.

### Endpoint thresholds are corpus- and use-case-specific

The selected thresholds operationalize distinct citation-risk states. Three or fewer citations within 2 years is a broader and more prevalent low-visibility state aligned with common journal citation windows. Zero citations within 3 years operationalizes complete short-term invisibility. Three or fewer citations within 3 years provides a longer-horizon low-visibility state, while more than 20 citations within 2 years represents an exploratory high-citation endpoint. Other journals, specialties, or research questions could define different absolute thresholds using the same framework; the key constraint is that endpoints should be prespecified, interpretable, and aligned with the intended question.

### Pooled performance is not journal-local validity

The journal-local diagnostic showed that pooled multi-journal performance should not be treated as evidence of local validity. When the pooled endpoint models were applied to held-out Journal C papers, PR-AUC, F1, and precision@10% were materially lower than pooled corpus-level summaries. Local thresholding and recalibration sometimes changed F1 or Brier score, but they did not consistently improve ranking or thresholded classification. For the sparse high-citation endpoint, threshold adjustment improved F1 without improving ranking, indicating a decision-boundary change rather than a better ordering of papers.

These findings separate three quantities that are often conflated in citation-prediction studies: ranking quality, calibration, and thresholded classification. A pooled model may detect corpus-level signal while still requiring local evaluation before its scores can be interpreted in a specific venue or specialty subcontext. Conversely, local thresholding may improve F1 without changing PR-AUC. Consistent with calibration-focused guidance in prediction modeling (Riley et al., 2016; Van Calster et al., 2019; Vollmer et al., 2020), pooled citation-prediction benchmarks should be interpreted as evidence of corpus-level signal, not as evidence of local validity, calibration, or deployment readiness.

### Limitations

These conclusions should be interpreted within four constraints. The study was internally validated within a seven-journal clinical gastroenterology corpus, without external validation in an independent journal set, specialty, or later publication era. The held-out test years were 2021 and 2022, so early citation trajectories may have been affected by COVID-era shifts in publication volume, topical attention, or citation behavior. A descriptive sensitivity analysis showed similar primary-endpoint prevalence across pre-pandemic and pandemic-era publication windows, reducing concern that the endpoint definition itself was driven by COVID-era prevalence shifts, but this does not rule out era-related changes in feature-outcome relationships.

The corpus was bounded by specialty but not citation-homogeneous. Masked journals differed in citation volume, reference-list availability, PubMed abstract coverage, and endpoint prevalence. Masked-journal characteristics are reported in **Online Resource 8**, and the curated-branch prevalence sensitivity is reported in **Online Resource 9**. In that sensitivity, excluding the curated Journal E/G branch increased low-citation endpoint prevalence and reduced high-citation prevalence, supporting the interpretation that endpoint frequencies were source-branch dependent. Because the primary analysis used the reference-observed cohort, uneven bibliography coverage may affect both feature availability and interpretation, particularly for reference-composition and reference-context features. The Journal E/G clinical-research curation branch also relied on publication-type and MeSH-based preprocessing with manual audit agreement of approximately 83%, introducing additional corpus-construction uncertainty. These issues limit generalizability beyond the masked seven-journal corpus.

Publication-time validity applied to study-specific feature construction, preprocessing, model fitting, validation, and endpoint labeling, but not to the pretraining history of externally trained scientific embedding models. SPECTER2 embeddings were used as frozen transfer representations over publication-time title and abstract text, but their pretraining may encode scientific-document structure influenced by papers or citation relationships from the analytic period. The practical consequence is that semantic features may encode information that would not have been available to a model trained exclusively on pre-publication data. This limitation is inherent to the use of any pretrained scientific language model in a temporally constrained prediction study and is not resolvable without retraining SPECTER2 on a temporally bounded corpus, which was outside the scope of this work. Results involving semantic feature families should therefore be interpreted with this constraint in mind.

The journal-local analysis was a diagnostic, not a deployment validation. Local event counts were small for some endpoints, including 9 held-out Journal C events for 0 citations within 3 years and 43 events for > 20 citations within 2 years, limiting precision for local performance comparisons. Citation-risk outcomes also measure visibility and attention, not scientific merit. The framework was not designed or validated as an editorial decision-support tool, and its outputs should not be used to screen, reject, or rank manuscripts without prospective validation, calibration assessment, governance, and human oversight.

## Conclusions

A publication-time-valid citation-prediction framework identified measurable signal for citation-risk outcomes in a bounded clinical specialty literature corpus. For the primary endpoint of ≤ 3 citations within 2 years, whole-document semantic embeddings modestly improved performance beyond the nonsemantic citation/reference/context baseline. Structure-aware feature families did not improve the primary endpoint but provided endpoint-specific information for selected secondary outcomes, most clearly by improving thresholded classification for complete noncitation. Author-history features showed standalone signal but did not add meaningful incremental value beyond citation, reference, and context features.

These models should be interpreted as tools for studying visibility and attention patterns, not as measures of scientific merit. Endpoint thresholds remain corpus- and use-case-specific. Pooled multi-journal performance should not be interpreted as journal-local validity without local evaluation, calibration assessment, and governance. Publication-time-valid citation-risk modeling may therefore provide a reproducible framework for studying how evidence becomes visible within bounded literature ecosystems, motivating future work across additional specialties, journal sets, publication eras, and downstream evidence-to-practice settings.

## Supplementary Material

Supplementary Files

This is a list of supplementary files associated with this preprint. Click to download.

- SupplementaryMaterials.pdf

## Figures and Tables

**Table 1:** Distribution of the final masked seven-journal analytic corpus by source and publication year, after article-type eligibility and source-record exclusions. The final columns show the number and percentage of papers with a parsed, non-empty reference list used for reference-observed analyses.

Source	2017	2018	2019	2020	2021	2022	Total	Reference observed *n*	Reference observed %
Journal A	248	200	204	276	203	311	1442	1112	77.1%
Journal B	162	171	169	186	137	166	991	986	99.5%
Journal C	314	444	381	458	318	300	2215	1763	79.6%
Journal D	324	268	385	492	422	475	2366	2198	92.9%
Journal E	146	156	146	217	173	117	955	899	94.1%
Journal F	115	143	150	191	145	135	879	875	99.5%
Journal G	91	76	94	142	100	73	576	576	100.0%
All	1400	1458	1529	1962	1498	1577	9424	8409	89.2%

**Table 2:** Endpoint-specific denominators and positive-label prevalence for the primary reference-observed cohort and the full analytic cohort. The primary endpoint is ≤ 3 citations within 2 years. Three-year endpoints use papers with three-year citation outcomes; two-year endpoints are restricted to papers with recovered yearly citation trajectories.

Cohort	Endpoint	Eligible n	Positive n	Prevalence
**Reference observed**	≤ 3 citations in 2 years	7988	3034	38.0%
**Reference observed**	0 citations in 3 years	8409	648	7.7%
**Reference observed**	≤ 1 citation in 3 years	8409	1204	14.3%
**Reference observed**	≤ 3 citations in 3 years	8409	1987	23.6%
**Reference observed**	> 20 citations in 2 years	7988	944	11.8%
**Full analytic cohort**	≤ 3 citations in 2 years	8711	3711	42.6%
**Full analytic cohort**	0 citations in 3 years	9424	1132	12.0%
**Full analytic cohort**	≤ 1 citation in 3 years	9424	1965	20.9%
**Full analytic cohort**	≤ 3 citations in 3 years	9424	2911	30.9%
**Full analytic cohort**	> 20 citations in 2 years	8711	948	10.9%

**Table 3:** Main endpoint comparisons in the reference-observed cohort. For each endpoint, the table reports the nonsemantic citation/reference/context baseline, the whole-document SPECTER2 semantic comparator, and the endpoint-specific retained family. Values are pooled held-out metrics in the reference-observed cohort. Metric triplets are PR-AUC / F1 / Precision@10%, where Precision@10% denotes precision among the top-ranked decile of held-out predictions. All family metric triplets include fold-stratified bootstrap 95% intervals.

Endpoint	Nonsemantic baseline PR-AUC/ F1/ Precision@10% (95% CI)	Whole-document semantic PR-AUC/ F1/ Precision@10% (95% CI)	Retained family	Retained PR-AUC/ F1/ Precision@10% (95% CI)
≤ 3 citations in 2 years	0.818 (0.799–0.837) / 0.722 (0.702–0.741) / 0.935 (0.912–0.970)	0.828 (0.809–0.847) / 0.735 (0.716–0.754) / 0.962 (0.935–0.981)	Whole-document semantic	0.828 (0.809–0.847) / 0.735 (0.716–0.754) / 0.962 (0.935–0.981)
0 citations in 3 years	0.436 (0.394–0.491) / 0.408 (0.363–0.451) / 0.434 (0.388–0.490)	0.472 (0.427–0.529) / 0.325 (0.285–0.367) / 0.458 (0.409–0.507)	Structure-aware content + context	0.472 (0.429–0.530) / 0.453 (0.413–0.494) / 0.458 (0.417–0.510)
≤ 3 citations in 3 years	0.858 (0.838–0.878) / 0.799 (0.777–0.820) / 0.937 (0.909–0.965)	0.860 (0.838–0.881) / 0.808 (0.788–0.827) / 0.941 (0.906–0.962)	Structure-aware content	0.866 (0.845–0.886) / 0.811 (0.791–0.830) / 0.948 (0.923–0.972)
> 20 citations in 2 years	0.452 (0.401–0.514) / 0.479 (0.435–0.525) / 0.460 (0.403–0.506)	0.464 (0.413–0.530) / 0.455 (0.417–0.491) / 0.491 (0.441–0.541)	Structure-aware contextual	0.509 (0.455–0.567) / 0.472 (0.428–0.515) / 0.491 (0.438–0.537)

## Data Availability

Analysis code, feature-construction scripts, model configuration files, and deidentified/masked derived analytic tables will be made available in a public repository upon publication. Raw bibliographic records subject to source restrictions will not be redistributed; instead, reproducible retrieval and processing scripts will be provided where permitted. Journal identities were masked throughout the manuscript to prevent reputational interpretation or implicit journal ranking; all corpus construction, feature derivation, temporal splitting, endpoint labeling, and performance reporting used stable masked identifiers. The masked-to-unmasked journal mapping is preserved in a controlled file held by the corresponding author and will be made available to editors and reviewers under confidentiality upon request. This mapping will not be included in the public archive. Researchers seeking to reproduce the full unmasked workflow may contact the corresponding author directly; any such disclosure will be governed by the data-use conditions of the originating OpenAlex and PubMed sources, which do not restrict journal-identity information.
